# Herbal-drug interaction induced rhabdomyolysis in a liposarcoma patient receiving trabectedin

**DOI:** 10.1186/1472-6882-13-199

**Published:** 2013-07-30

**Authors:** Sabino Strippoli, Vito Lorusso, Anna Albano, Michele Guida

**Affiliations:** 1 Medical Oncology Department National Cancer Research Centre “Giovanni Paolo II”, Bari, Italy; 2 National Cancer Research Centre “Giovanni Paolo II”, Via Orazio Flacco, Bari, 65. 70124, Italy

**Keywords:** Trabectedin related rhabdomyolysis, Liposarcomas, Drug-drug interactions, Chokeberry (*Aronia melanocarpa*)-drugs interaction

## Abstract

**Background:**

Rhabdomyolysis is an uncommon side effect of trabectedin which is used for the second line therapy of metastatic sarcoma after anthracycline and ifosfamide failure. This side effect may be due to pharmacokinetic interactions caused by shared mechanisms of metabolism involving the cytochrome P450 (CYP) system in the liver. Here, for the first time in literature, we describe the unexpected onset of heavy toxicity, including rhabdomyolysis, after the fourth course of trabectedin in a patient with retroperitoneal liposarcoma who at the same time was taking an alternative herbal medicine suspected of triggering this adverse event.

**Case presentation:**

This is the case of a 56 year old Caucasian man affected by a relapsed de-differentiated liposarcoma who, after the fourth cycle of second-line chemotherapy with trabectedin, complained of sudden weakness, difficulty walking and diffuse muscle pain necessitating complete bed rest. Upon admission to our ward the patient showed grade (G) 4 pancytopenia and a marked increase in liver lytic enzymes, serum levels of myoglobin, creatine phosphokinase (CPK) and lactate dehydrogenase. No cardiac or kidney function injuries were present. Based on these clinical and laboratory features, our conclusive diagnosis was of rhabdomyolysis induced by trabectedin.

The patient did not report any trauma or muscular overexertion and no co-morbidities were present. He had not received any drugs during treatment with trabectedin, but upon further questioning the patient informed us he had been taking a folk medicine preparation of chokeberry (*Aronia melanocarpa*) daily during the last course of trabectedin and in the 2 subsequent weeks.

One week after hospitalization and cessation of intake of chokeberry extract, CPK and other markers of myolysis slowly returned to standard range, and the patient noted a progressive recovery of muscle strength.

The patient was discharged on day 14 when a blood transfusion and parenteral hydration gradually lowered general toxicity. Progressive mobilization of the patient was obtained as well as a complete normalization of the laboratory findings.

**Conclusions:**

The level of evidence of drug interaction leading to the adverse event observed in our patient was 2 (probable). Thus our case underlines the importance of understanding rare treatment-related toxicities such as trabectedin-induced rhabdomyolysis and the possible role of the drug-drug interactions in the pathogenesis of this rare side effect. Furthermore, this report draws attention to a potential problem of particular concern, that of nutritional supplements and complementary and alternative drug interactions. These are not widely recognized and can cause treatment failure.

## Background

Despite aggressive surgery and adjuvant radiotherapy, more than half of patients with soft tissue sarcoma develop a locally advanced or metastatic disease [1]. Unfortunately there are very few active drugs for these patients. By far the most relevant change in this scenario is trabectedin, a drug isolated from the marine microorganism *Ecteinascidia turbinate*. Trabectedin was approved in Europe in 2007 as a single agent for second-line therapy of soft tissue sarcomas, and its activity is mainly relevant in liposarcoma and leiomyosarcoma [2].

While adverse reactions to trabectedin treatment such as neutropenia and liver function test alterations are well-known, more education and data are needed on some of the rare side effects mediated by this drug, such as the potential induction of rhabdomyolysis. A recent comprehensive safety analysis confirmed the uncommon occurrence of this side effect and also pointed out the need to avoid pharmacokinetic interactions with concomitant drugs caused by shared mechanisms of metabolism by the cytochrome P450 (CYP) system in the liver [3].

We describe here the unexpected onset of heavy toxicity including rhabdomyolysis after the fourth course of trabectedin in a patient with retroperitoneal liposarcoma who at the same time was taking an alternative herbal medicine suspected of triggering this adverse event. This report calls attention to a potential problem of particular concern, that of herbal-drug interaction. This problem is not widely recognized and can cause treatment failure in patients without an immediately obvious clinical modification.

## Case presentation

This is the case of a 56 year old Caucasian man from Montenegro whose cancer history began in April 2009 after the appearance of progressive abdominal pain due to a retroperitoneum mass measuring 5 × 8 cm. The patient’s past medical record showed no relevant illness. He was an ex heavy smoker and did not drink alcohol or use illicit drugs. In May 2009 he underwent surgical removal of the abdominal mass achieving a complete margin negative (R0) resection. Histological findings of lesion specimens highlighted a low-grade de-differentiated liposarcoma. He then began follow-up. In November 2011 a computed tomography (CT) scan detected a new retroperitoneal lesion measuring 5 × 10 cm whose imaging features clearly suggested relapsed liposarcoma. He therefore underwent another radical surgery and the second histological examination supported the diagnosis of G2 de-differentiated liposarcoma with myxoid areas. Fluorescence in situ hybridization (FISH) analysis for oncogene murine double minute 2 (MDM2) status highlighted the amplification as highly specific diagnostic markers [4].

After only three months, a new recurrence occurred and owing to the brief relapse-free time he was not considered suitable for additional surgery. Accordingly, he was treated with chemotherapy including three cycles of doxorubicin followed by three cycles of ifosfamide. In spite of chemotherapy, the lesion progressed rapidly reaching 16 × 10 cm and extending into the pre-aortic seat of the retro-peritoneum space. In April 2012 the patient was referred to our Institute and underwent second line chemotherapy with trabectedin at a dose of 1.5 mg/m [2], administered as a 24-hour continuous intravenous infusion repeated every 21 days. Intravenous premedication was normally used before trabectedin administration and included dexamethasone 12 mg, palenosetron 250 mcg and chlorphenamine maleate 10 mg. Due to the previous chemotherapy treatment, we decided to use prophylactic peg-G-CSF (granulocytes colony stimulating factor) administered the day after chemotherapy. Chemotherapy was well tolerated for the first three courses, and no abnormalities regarding hematological, liver and muscle enzyme levels were present. At this time, the patient’s Eastern Cooperative Oncology Group (ECOG) performance status was 0 and stable disease was documented by an abdominal CT scan examination. Due to no alternative therapeutic options, we decided to continue chemotherapy with trabectedin. Two weeks after the fourth cycle of chemotherapy, the patient began to complain of sudden weakness, difficulty walking and diffuse muscle pain. Some days after he was confined to complete bed rest as the muscle pain became unbearable. For these reasons, the patient was urgently admitted to our ward and parenteral hydration and symptomatic therapy were initiated. Laboratory tests found G4 pancytopenia and a marked increase in liver lytic enzymes. At the same time, serum levels of myoglobin, creatine phosphokinase (CPK) and lactate dehydrogenase (LDH) showed an increase respectively of eighteen, eight and four times over the upper normal range value (Table 1). The absence of abnormalities on electrocardiogram and echocardiography ruled out cardiac etiology for the increased serum level of these enzymes. No kidney function injury was present. Due to these clinical and laboratory features, our conclusive diagnosis was of rhabdomyolysis induced by trabectedin.

**Table 1:** Behavior of laboratory parameters related to rhabdomyolysis starting from the first day of chemotherapy

**Laboratory test**	**Normal range**	**Day 1**	**Day 13**	**Day 15**	**Day 17**	**Day 20**
White blood cell count	4-10 × 10^3^/μl	3,6	3,2	2,5	3,6	2,9
Absolute neutrophils	1,7-7,6 × 10^3^/μl	1,6	2,3	1,3	1,1	1,6
Hemoglobin	13,5-17,5 g/dl	12,2	7,5	8,8	9,1	9,3
Platelets	150-450 ×10^3^/μl	335	77	162	367	389
ALT	0-41 U/L	36	449	294	89	44
AST	0-40 U/L	34	504	176	31	21
LDH	135-225 U/L	165	986	543	286	201
Myoglobin	28-72 ng/ml	Not tested	1349	981	148,5	83,32
CPK	0-190 U/L	Not tested	1619	1126	217	107

As the patient had this unusual muscular side effect after the fourth cycle of trabectedin, we explored possible sources that could trigger the potential trabectedin-induced rhabdomyolysis. He did not report any trauma or muscular overexertion. Due to the absence of co-morbidity, the patient had not received any other drugs during treatment with trabectedin. After further questioning in a non-threatening fashion, the patient informed us that he had been taking a commercial folk medicine preparation of chokeberry juice (*Aronia melanocarpa*) daily during the last course of trabectedin and in the 2 weeks after.

One week after hospitalization and stopping intake of chokeberry extract, CPK and other markers of myolysis slowly returned to standard range and the patient felt a progressive recovery of muscle strength.

The patient was discharged on day 14 when blood transfusion and parenteral hydration gradually reduced general toxicity. A progressive mobilization of the patient was obtained as well as a complete normalization of the laboratory findings (Table 1).

## Discussion and conclusions

Among mesenchymal tumors, liposarcoma has proven to be the most sensitive to the marine alkaloid trabectedin [2]. This antitumor agent is used in second line therapy after anthracycline and ifosfamide failure. In this context, trabectedin acquires a central role in retroperitoneum liposarcoma which relapses very frequently and exhibits low response to few drugs. The most common side effects of trabectedin include myelotoxicity and liver toxicity, mainly in the form of an increase of transaminase. Rhabdomyolysis is an uncommon side effect of trabectedin with an incidence of 0.5-0.7% [3,5]. Nevertheless, it can have an important impact on the morbidity and mortality of patients, and lead to renal failure due to serum release of toxic muscle cell components. Even if multivariate analysis does not identify any predictor factor of rhabdomyolysis developing, it is mandatory to avoid conditions which could trigger this occurrence such as direct trauma, seizures, extreme exertion, body-temperature extremes, muscle hypoxia, infections (sepsis and severe pneumonia) and endocrine disorders (hypothyroidism). Rhabdomyolysis is not a cumulative adverse effect of trabectedin because the majority of cases happen during the first 3 cycles, but the mechanism by which it may be caused is unknown. It is possible to deduce from safety analysis and case reports that a relevant risk issue is pharmacodynamic and pharmacokinetic drug interaction [6]. Beyond the pharmacodynamic interaction with substances of abuse such as alcohol and cocaine or medications like statins, concomitant treatments with drugs exerting a CYP inhibitor activity can increase serum trabectedin levels leading to toxicity. This is because trabectedin is eliminated through hepatic metabolism and CYP3A4 is the principal responsible enzyme mediating its degradation [7]. As known, cancer patients are likely to experience drug interactions due to the increased number and types of chemotherapeutic drugs, their combinations in oncology practice and their narrow therapeutic index, the achievement of a prolonged life expectancy, and the common use of a broad range of ancillary medications to prevent and/or treat cancer-related syndromes and treatment-induced toxicity [8]. In some cases, a drug interaction may result from an unexpected source, such as herbs or vitamins, which most patients do not consider to be drugs. Given the lack of effectiveness, many cancer patients treated with conventional therapies also try 'alternative' cancer treatments even when beneficial effects have not been proven [9]. There are many challenges related to alternative medication as a treatment for cancer, not the least toxicity related to the interaction with conventional chemotherapy. Despite the increasing use of these alternative medications among cancer patients, the breadth of their side effects is not always well known and is still seldom addressed, therefore these patients risk being more harmed than helped by these compounds [10,11]. In Table 2 the most common herbal medicines involved in pharmacokinetic interactions with chemotherapeutic drugs via CYP are reported.

**Table 2:** An overview of the most common herbal medicines involved in pharmacokinetic CYP interactions with chemotherapeutic drugs

**Herb**	**Indication**	**CYP involvment**	**Effect on metabolism**	**Reference**
*Ginko biloba*	Psychostimulant	2 D6	inhibition	[12]
		3 A4		
*Aloe vera*	Antioxidant	3 A	inhibition	[13]
	Antiproliferative			
	Immunostimulant	2C9		
*Green tea*	Immunostimulant	3 A	inhibition	[14]
*Saint John*’*s Wort*	Antidepressant	3 A4	inhibition	[15]
		2 C9		
		1 A2		
		1 B1		
		2 D6		
*Ginseng*	Psychostimulant	3 A	inhibition	[16]
		2 C9		
	Immunostimulant	2 C19		
		2 d6		
*Echinacea*	Immunostimulant	3 A	inhibition	[17]
		2 D6		
*Garlic*	Antioxidant	3 A	inhibition	[18]
	Immunostimulant	2 C9		
	Antiplatelet activity	2 C19		

In our patient this “natural foe” was chokeberry (*Aronia melanocarpa*) extract derived from the black fruit of this shrub. This berry has a long tradition in European and North American folk medicine [19]. It is a relatively concentrated source of flavonoids such as quercetin, and is also reported to strongly inhibit CYP3A4 activity in the liver. Consequently it is capable of increasing the bioavailability of different chemotherapeutic agents [20]. Furthermore, the polyphenol anthocyanins in these berries are proven to have antioxidant properties which positively influence several risk factors for cardiovascular disease as well as anti-proliferative or protective effects against colon cancer [21,22]. Several other substances such as sorbitol and polyphenols like laetrile advocated by some as a “cure” or a “preventative” for cancer are burdened by considerable doubt about their safety, and there are no proven beneficial effects for cancer patients [7].

From a clinical-pharmacological point of view, due to the evidence concerning decreased activities of enzymatic markers of CYP after forced feeding of chokeberry juice [23], the adverse event observed in our patient was secondary to the herbal-chemotherapy interaction with a level of evidence of 2 (probable) [24]. However, there are limitations that deserve to be noted which should be appropriately handled in future studies: the idiosyncratic nature of trabectedin-related rhabdomyolysis; the patient’s pharmacokinetic parameters which have been altered by the cancer and the therapy itself; and the conceivable re-challenge with trabectedin after this side effect [25]. Moreover, as the patient took a commercial preparation of chokeberry juice which did not provide information on the amounts of its compounds, it cannot be excluded that other interactions might have occurred such as the inhibition of transport mechanisms, the activation of the death receptor and other unknown mechanisms. Thus these potential interactions could occur not only with trabectedin but also with other chemotherapies. The development of medication databases linked to electronic screening programs is a useful tool that could help health professionals to identify dangerous herbal-drug combinations in the oncology setting [26].

In conclusion, our case underlines the importance of understanding rare treatment-related toxicities such as trabectedin-induced rhabdomyolysis and the possible role of the drug-drug interactions in the pathogenesis of this rare trabectedin side effect. Furthermore, in the presence of unexpected toxicity during conventional anticancer treatment, the simultaneous use by the patient of alternative drugs should be suspected. So a thorough medication history, enquiring about nutritional supplements and over-the-counter drugs, may help raise some red flags about potential interactions with chemotherapy.

## Consent

Written informed consent was obtained from the patient for the publication of this report.

## Abbreviations

CYP: Cytochrome P450; CT: Computed tomography; MDM2: Oncogene murine double minute 2; peg-G-CSF: Pegylated granulocytes colony stimulating factor; ECOG: Eastern Cooperative Oncology Group; CPK: Creatine phosphokinase; LDH: Lactate dehydrogenase; ALT: Alanine transaminase; AST: Aspartate transaminase.

## Competing interests

The authors declare that they have no competing interests.

## Authors’ contributions

SS, MG and VL treated and clinically evaluated the patient, SS and MG drafted the manuscript and were principle authors of the paper, VL and AA contributed to draft the manuscript. All authors contributed to manuscript revision and approved the final manuscript.

## Pre-publication history

The pre-publication history for this paper can be accessed here:

http://www.biomedcentral.com/1472-6882/13/199/prepub
